# Multi-Omics Research on Angina Pectoris: A Novel Perspective

**DOI:** 10.14336/AD.2024.1298

**Published:** 2025-12-06

**Authors:** Haiyang Chen, Lijun Zhang, Meiyan Liu, Yanwei Li, Yunpeng Chi

**Affiliations:** ^1^Department of Psycho-cardiology, Beijing Anzhen Hospital, Capital Medical University, Beijing, China.; ^2^School of Clinical Medicine, Henan University, Kaifeng, China.

**Keywords:** Angina pectoris, multi-omics, biomarkers, systems biology

## Abstract

Angina pectoris (AP), a clinical syndrome characterized by paroxysmal chest pain, is caused by insufficient blood supply to the coronary arteries and sudden temporary myocardial ischemia and hypoxia. Long-term AP typically induces other cardiovascular events, including myocardial infarction and heart failure, posing a serious threat to patient safety. However, AP’s complex pathological mechanisms and developmental processes introduce significant challenges in the rapid diagnosis and accurate treatment of its different subtypes, including stable angina pectoris (SAP), unstable angina pectoris (UAP), and variant angina pectoris (VAP). Omics research has contributed significantly to revealing the pathological mechanisms of various diseases with the rapid development of high-throughput sequencing approaches. The application of multi-omics approaches effectively interprets systematic information on diseases from the perspective of genes, RNAs, proteins, and metabolites. Integrating multi-omics research introduces novel avenues for identifying biomarkers to distinguish different AP subtypes. This study reviewed articles related to multi-omics and AP to elaborate on the research progress in multi-omics approaches (including genomics, transcriptomics, proteomics, and metabolomics), summarized their applications in screening biomarkers employed to discriminate multiple AP subtypes, and delineated integration methods for multi-omics approaches. Finally, we discussed the advantages and disadvantages of applying a single-omics approach in distinguishing diverse AP subtypes. Our review demonstrated that the integration of multi-omics technologies is preferable for quick and precise diagnosis of the three AP types, namely SAP, UAP, and VAP.

## 1. Introduction

Aging Coronary heart disease (CHD) has gradually transformed into a primary fatal disease with the aging worldwide population [1]. The World Heart Federation forecasted that the mortality of patients with CHD is expected to reach 23.6 million worldwide, posing serious threats to life and safety, particularly in elderly individuals [2]. Angina pectoris (AP), a common clinical symptom of CHD, is predominantly characterized by paroxysmal chest pain, which is caused by myocardial ischemia and hypoxia [3, 4]. Clinically, AP is classified into three types: stable angina pectoris (SAP), unstable angina pectoris (UAP), and variant angina pectoris (VAP). SAP, the most common AP subtype and one of the chronic diseases, is typically regarded as a warning signal or initial event of the majority of myocardial ischemia cases, with a period of clinical manifestation of 1-3 months [5-7]. UAP, a significantly aggravated AP, manifests as a heterogeneous syndrome of cardiac injury and is frequently considered a predecessor to acute myocardial infarction (AMI) and sudden death [8, 9]. VAP, also termed vasospastic angina pectoris, is caused by coronary vasospasm with clinical symptoms of pectoralgia accompanied by ST segment abnormalities on electrocardiography, whose occurrence is also closely related to AMI and sudden cardiac death [10-12]. Percutaneous coronary intervention contributes to improving most AP cases; nevertheless, specific medication treatment strategies for SAP, UAP, and VAP slightly differ. Lipid-lowering and antiplatelet medications can be considered for SAP treatment, in addition to conventional antianginal drugs (including β-blockers, nitrates, and calcium channel blockers) [13, 14]. For UAP therapy, other anticoagulants are normally added based on the above-mentioned therapeutic schedule since the severity of UAP exceeds that of SAP [15]. For VAP therapy, angiotensin-converting enzyme inhibitors (ACEIs) and angiotensin receptor blockers (ARBs) are additionally administered [16]. Differentiating patients with AP into the above three subtypes is crucial to achieving precise treatment owing to the differences in the administered clinical medications. Profound side effects may result if imprecise medications are administered, complicating clinical treatment tasks and posing a threat to patient safety. For example, excessive antiplatelet drugs or anticoagulants may increase the risk of bleeding, and inappropriate administration of ACEIs or ARBs may result in hypotension, hyperkalemia, vascular edema, and renal function deterioration [17, 18]. Unfortunately, current classification approaches for the three AP subtypes are confined to the long-term observation of clinical manifestations, such as chest pain characteristics, duration and frequency of onset, and whether a resting state or nitroglycerin administration will relieve symptoms, in addition to judgment based on results of electrocardiograms and coronary angiography, which are time-consuming, expensive, and may lead to misdiagnoses [19, 20]. Therefore, developing novel strategies for the rapid and accurate classification of AP is imperative.

Omics, the systematic study of various research objects, particularly biomolecules, aims to comprehensively characterize and quantify the structure, function, and dynamics of biological molecular pools in organisms [21]. Progress in omics technology has contributed to medical research, particularly in revealing pathological mechanisms and identifying biomarkers during the entire process of disorders [22, 23]. While various approaches have been employed to identify biomolecules, omics technologies achieve more extensive and rapid qualitative characterization and quantification of biomolecular data [24]. In contrast to the traditional diagnostic methods for AP classification (including medical history inquiry, myocardial injury markers detection, and electrocardiogram and coronary angiography examination), multi-omics technologies, namely genomics, transcriptomics, proteomics, and metabolomics, contribute to improving the sensitivity and specificity of diagnosis and achieving personalized diagnosis and precision medicine by tracing comprehensive and multidimensional biological information in different pathological stages of CHD from the perspective of genes, proteins, and metabolites, respectively [25]. For instance, the integration of proteomics and metabolomics has indicated the involvement of multiple metabolic pathways, including energy metabolism, amino acid metabolism, and vascular smooth muscle contraction, in the occurrence of myocardial ischemia, which is an early stage of CHD [26]. A recent study reported 12 types of urinary proteins with differential expression as candidate biomarkers possessing high specificity and sensitivity for AMI diagnosis, which is the middle stage of CHD [27]. The combination of plasma proteomics and transcriptomics effectively identified six biomarkers for accurately distinguishing heart failure, which is frequently the terminal stage of CHD [28]. Therefore, multi-omics technologies are expected to facilitate a comprehensive and penetrative understanding of phases and types of diseases through the analysis of multifaceted biomolecular information.

The onset and progress of AP, a typical symptom of cardiovascular diseases, is determined by gene phenotypes while being strongly affected by multiple other factors, such as lifestyle, dietary habits, and mental stress [29, 30]. The application of multi-omics in recent years has led to great achievements in identifying biomarkers and therapeutic targets for diverse diseases, including cardiovascular and cerebrovascular disorders and cancers [31-34]. Therefore, in this study, we reviewed current research on the application of multi-omics strategies in AP. We further discussed the possibility of selecting potential candidates using multi-omics technologies to effectively discriminate among three AP subtypes, thereby achieving precision medicine.

## 2. Inclusion and exclusion criteria

We searched relevant literature on the use of omics technologies in angina in databases, including the Web of Science, PubMed, and Scopus databases, using “angina”, “genomics”, “transcriptomics”, “proteomics”, and “metabolomics” as search terms. Articles that did not adopt omics technology and review articles were excluded from the analysis.

## 3. Genomics

Genomics is the interdisciplinary study of the qualification and quantification of all genes and the comparison of differential genes and focuses on investigating genome structure, function, evolution, localization, editing, and impact on organisms [35]. Genomics rapidly progressed after being first proposed, with its greatest achievement being the Human Genome Project (HGP) completed in the early 21st century [36]. High-throughput DNA sequencing technology, also termed next-generation sequencing (NGS), has gradually become an efficient tool with advancements in gene sequencing approaches. NGS technology enables the simultaneous sequencing of hundreds of thousands to millions of nucleic acid molecules, whose fundamental principle involves assembling all sequences generated by hundreds of short base segments fragmented from the genome [37]. NGS adoption has significantly advanced the medical field; for instance, NGS rapidly discriminates heterogeneous genes in emerging diseases during gene sequencing diagnosis to fulfill accurate medication requirements and avoid substantial injury to patients. Therefore, genetic biomarkers for successful discrimination of the three AP subtypes were sought by further exploring the genetic differences between them and those of normal individuals in the following section. In addition, the potential biomarkers or therapeutic targets for identifying the different AP subtypes of genomics studies are summarized in Table 1.

**Table 1:** Potential biomarkers or therapeutic targets for identifying different AP subtypes in genomics studies.

AP subtypes	Samples	Platforms	Biomarkers/therapeutic targets	Datasets	Refs
SAP	Neutrophils in peripheral blood	Illumina Hiseq 2000	H3k4me1/2 and H3k27ac	RNA-Seq and ChIP-Seq data	[39]
SAP	Blood	Affymetrix GeneChip® Human Mapping 6.0 Array	*rs17300022*, *rs6904106*, *rs17177818*, *rs2248165*, *rs2477539*, *rs16865681*, *rs2396058*, *rs4753663*, *rs4082252*, *rs12862206, rs6932*, and *rs6780676*, in or nearby GNA12, NMBR, CUL3, SFMBT2, SESN3, SLC22A25, EFBN2, and SEC62	LURIC and CARDIoGRAM datasets	[41]
SAP	Peripheral blood	PCR-LDR sequencing	*rs10811656*, located in the *9p21.3* locus of chromosome	dbSNP database	[42]
UAP	Peripheral venous blood samples	Illumina Hiseq 2000	Descended diversity of T-cell receptor and B-cell receptor repertoire	Human IGH database	[43, 44]
UAP	Blood	ABI PRISM 377 gene sequencers	Gene loci in chromosome 2q36-q37.3, 3q26-q27, and 20q11-q13	ABI PRISM Human Linkage Mapping Set	[45]
UAP	Blood	ABI 3100 capillary-sequencing machine	FcγRIIa R/R131 genotype	/	[46]
UAP	Peripheral blood leukocytes	LightScanner 96 system	Variation of *rs2107595* in the HDAC9 gene	/	[48]
UAP	Peripheral blood leukocytes	HumanCoreExome-24 Version 1-0A microarray	Variation of *rs17609940* in the ANKS1A gene	/	[47]
UAP	Whole blood	Illumina Infinium Human Methylation450K BeadChip	Methylation of multiple cytosine-phosphate-guanine islands located in ATP2B2, CASR, GUCA1B, PTPRN2, and HPCAL1	ARIC, CHS, EPICOR, WHI-EMPC, InCHIANTI, and KORA data and FHS DNA methylation, genotype, NAS DNA methylation, and WHI-BAA23 DNA methylation datasets	[49]
VAP	Blood leukocytes	GeneChip DNA Analysis	SNP of *rs10498345* located on chromosome 14q21.1	HapMap data	[50]
VAP	Peripheral blood	ABI PRISM 7900HT Sequence Detection System	Mutation of Glu298Asp in the endothelial nitric oxide synthase gene	/	[51]
VAP	Peripheral blood mononuclear cells	GeneScan technique	State of alpha2CDel322-325 allele carrier and beta2Gln27 allele homozygote	/	[52]

### 3.1. Genomics in SAP

Clarification of genetic risk factors is instrumental in revealing the pathological mechanisms and identifying novel therapeutic targets, which are essential for the timely ameliorating of preventative and therapeutic regimens for various disorders [38]. Genomic variation permeates the entire period of CHD, beginning from SAP, in which alterations of inflammation-related and angiogenesis-related genes play a crucial role [6]. For example, H3k4me1/2 and H3k27ac were identified as novel therapeutic targets for electro-acupuncture-treated SAP after employing NGS to sequence gene expression profiles of neutrophils in peripheral blood [39].

NGS and genome-wide association studies (GWAS) have identified genetic factors (single nucleotide polymorphisms, SNPs) related to complex diseases and genetic genes (namely, gene loci) related to disease occurrence, development, and treatment [40]. After genome-wide sequencing of coronary plaque, twelve SNPs, namely, *rs17300022*, *rs6904106*, *rs17177818*, *rs2248165*, *rs2477539*, *rs16865681*, *rs2396058*, *rs4753663*, *rs4082252*, *rs6932*, *rs12862206*, and *rs6780676*, in or nearby eight genes, including GNA12, NMBR, SFMBT2, CUL3, SESN3, SLC22A25, EFBN2, and SEC62, were identified as candidates for the diagnosis of European patients with SAP with a coronary plaque [41]. Meanwhile, the presence of one SNP, *rs10811656*, located in the 9p21.3 locus of peripheral blood chromosome, has been confirmed as highly relevant to SAP in Asian regions, particularly in Chinese populations [42].

### 3.2. Genomics in UAP

High-throughput sequencing of T-cell receptor (TCR) and B-cell receptor (BCR) repertoires revealed a decline in the multiplicity of TCR and BCR in the UAP samples compared to normal samples, indicating extensive involvement of immunoreactions in the generation of UAP, both of whose descended diversity may be regarded as the symbols for UAP [43, 44]. Genome-wide scanning revealed that variations in three gene loci located on chromosome 2q36-q37.3, chromosome 3q26-q27, and chromosome 20q11-q13 indicated high susceptibility to the emergence of UAP in patients with positive family history for CHD [45]. Moreover, the risk of UAP was effectively predicted by determining the polymorphism of the FcγRIIa R131 gene in patients, namely whether they carry the FcγRIIa R/R131 genotype [46]. In addition, the SNP in certain genes has been identified as biomarkers for UAP. For example, GWAS revealed the variation of *rs2107595* in the HDAC9 gene and *rs17609940* in the ANKS1A gene were eminently associated with UAP [47, 48]. Apart from SNPs induced by the alteration of bases, epigenetic modification of genes plays an essential role in cardiovascular event occurrence. Methylation of multiple cytosine-phosphate-guanine islands located in several genes originating from peripheral blood DNA, including ATP2B2, CASR, GUCA1B, PTPRN2, and HPCAL1, prompted transcriptional silencing, which was an effective tool for identifying UAP [49].

### 3.3. Genomics in VAP

SNP *rs10498345* located on chromosome 14q21.1 and mutation of Glu298Asp in the endothelial nitric oxide synthase gene have been confirmed to affect vasoconstriction in response to acetylcholine, all of which are closely related to coronary spasm-induced angina [50, 51]. In addition, polymorphisms of the adrenergic receptor gene, namely the alpha2CDel322-325 allele carrier and beta2Gln27 allele homozygote, were regarded as novel genetic risk factors for VAP [52]. However, there were still bottlenecks in genomics owing to the splicing problem of repetitive sequences while using diverse sequencing approaches, and gene mutations are only a partial inducement for diseases. Therefore, more comprehensive information on disease development can only be acquired by integrating multiple omics strategies, including transcriptomics, proteomics, and metabolomic.

## 4. Transcriptomics

Transcription, the first step in the central genetic dogma, is the process of transforming genetic information into different recognizable phenotypes [53]. Transcriptomics, a useful tool for studying phenotypes and gene function, aims to investigate the summation of transcribed RNAs [i.e., messenger RNAs, transfer RNAs, ribosomal RNAs, and long non-coding RNAs (lncRNAs)] from genes and the law of transcriptional regulation in cells [54]. Transcribed phenotypes are limited by time and space, with diversity in transcriptional products of the same cells during different growth stages and environments [55]. Therefore, developing an approach to effectively and comprehensively inform the total transcriptional products is necessary to facilitate disease understanding, particularly regarding the differentiation of disease subtypes. Moreover, with the help of multiple sequencing technologies, such as microarray analysis, transcriptomics enables the classification and quantification of each transcript under different pathological processes [56]. The coding transcripts, as one type of RNA transcript, are further translated into proteins, most of which are related to the inflammatory response and exhibit a definite role in the discrimination of AP subtypes [57, 58]. Another type of RNA transcript, a noncoding transcript, reversely influences the expression of genes regulating the pathological process of AP [59]. However, the current studies about the effects of the non-coding transcript on AP are insufficient. Therefore, we focused on the application of diverse sequencing technologies, particularly microarray analysis, to determine non-coding transcripts, including microRNAs (miRNAs) and lncRNAs, in SAP and UAP in the following sections. The potential biomarkers or therapeutic targets for identifying different AP subtypes in transcriptomic studies are summarized in Table 2.

**Table 2:** Potential biomarkers or therapeutic targets for identifying different AP subtypes in transcriptomics studies.

AP subtypes	Samples	Platforms	Biomarkers/therapeutic targets	Datasets	Refs
SAP	Peripheral venous blood	Illumina HiSeq 2500 platform	miR-7109-3p, miR-6515-3p, miR-1273g-3p, miR-20b-5p, miR-6793-5p, miR-142-3p, miR-1-3p, and miR-30b-5p	miRanda, MicroCosm, and Targetscan databases	[60]
SAP	Plasma	Agilent Human lncRNA + mRNA Array V4.0	lncRNAs NR_037652.1, ENST00000607654.1, ENST00000589524.1 and uc004bhb.3	GENCODE/ENSEMBL, LNCipedia, the Human LincRNA Catalog, the ncRNA Expression Database, RefSeq, the University of California, Santa Cruz, and the Chen Ruisheng laboratory (Institute of Biophysics, Chinese Academy of Science) databases	[59]
UAP	Peripheral venous blood	Agilent miRNA microarray system	Down-regulation of miR-1202, miR-1207-5p, and miR-1225-5p, up-regulation of miR-3162-3p, especially for miR-3162-3p, miR-1225-5p, and miR-1207-5p	Mirbase, Miranda, and Targetscan databases	[61]
UAP	Plasma	Taqman low-density miRNA array	Elevation of miR-106b/25 cluster, miR-17/92a cluster, miR-21/590-5p family, miR-126*, and miR-451	Gene Expression Omnibus database	[62]

### 4.1 Transcriptomics in SAP

Multiple miRNAs and lncRNAs serving as SAP biomarkers were identified by combining various microarray analysis approaches and qRT-PCR validation. The upregulation of diverse miRNAs, including miR-7109-3p, miR-6515-3p, miR-1273g-3p, miR-20b-5p, miR-6793-5p, miR-142-3p, miR-1-3p, and miR-30b-5p, effectively distinguishes SAP and UAP from normal samples [60]. Moreover, four novel lncRNA markers of SAP among Uyghur patients, including NR_037652.1, ENST00000607654.1, ENST00000589524.1, and uc004 bhb.3, were screened by combining Agilent microarray analysis and qRT-PCR validation [59].

### 4.2 Transcriptomics in UAP

Microarray-based miRNA chip analysis and qRT-PCR revealed that the combination of three downregulated genes, including miR-1202, miR-1207-5p, and miR-1225-5p, and one upregulated gene, including miR-3162-3p was considered the most significant candidate for the rapid and accurate diagnosis of patients with UAP [61]. The combination of TaqMan miRNA array and co-expression network analyses revealed that significant elevations of the miR-106b/25 cluster, miR-17/92a cluster, miR-21/590-5p family, miR-126*, and miR-451 were biomarkers of UAP occurrence [62].

## 5. Proteomics

Proteins, as the final transcribed and translated gene product, are frequently not the direct products of genes, that is, the number of proteins is generally greater than that of genes owing to the difference in shear patterns and post-translational modifications [63]. Moreover, proteomics is closer to disease phenotype or symptoms than genomics [64]. Proteomic studies provide a material basis for the laws of life activities and a theoretical basis and solutions for elucidating and conquering various disease mechanisms [65]. Therefore, proteomics has gradually developed into an attractive research area for identifying protein biomarkers of multiple diseases with diverse phenotypes, particularly different AP phenotypes. In the following section, we primarily focused on the application of proteomic analysis approaches in searching for biomarkers for the discrimination of SAP and UAP; however, VAP was not included because the relevant references were inadequate. Potential biomarkers or therapeutic targets for identifying different AP subtypes in proteomic studies are summarized in Table 3.

**Table 3:** Potential biomarkers or therapeutic targets for identifying different AP subtypes in proteomics studies.

AP subtypes	Samples	Platforms	Biomarkers/therapeutic targets	Datasets	Refs
SAP	Plasma	LC-MS/MS	F10, MST1, SERPINA3, CPN2, LUM, ORM2, ACTG1, and NAGLU	SRM/MRM transitions database	[66]
SAP	Platelets	MS/MS	Profilin-1, calpain, α-soluble NSF attachment protein, thrombospondin, ubiquitin-like modifier-activating enzyme 1, protein-L-isoaspartate-(D-aspartate) O-methyltransferase, nucleoside diphosphate kinase B, heat shock 70 kDa protein 5, and anti-stress induced phosphoprotein 1	/	[67]
SAP	Platelets	MS/MS	2-oxoglutarate dehydrogenase, lactate dehydrogenase, gamma-actin, coronin 1B, pleckstrin, and proteasome subunit type 8	SwissProt and NCBInr protein databases	[68]
SAP	Angioplasty balloons	LC-MS/MS	206 kinds of differential proteins	PRIDE database	[69]
SAP	Plasma	UltiMate 3000 nanoLC MS	Apolipoprotein A1	MASCOT database	[70]
SAP	Whole blood	MS/MS	Fibrinogen, fetuin-B, keratin 9, pro-apolipoprotein, and fibrinogen	MASCOT database	[71]
UAP	Serum	SELDI-TOF-MS	Hepcidin-25, MRP8, SAA, B2M, MPO, and βTG	/	[72]
UAP	Venous blood	Siemens BNII instrument	Reduction of serum complemental C1q	/	[73]
UAP	Plasma	Perkin Elmer SpotArray Enterprise non-contact arrayer	D-dimer, hepatocyte growth factor, CXCL9/MIG, platelet factor 4/CXCL4, CTACK, C-6 Kine, follistatin, FGF-7, PAI-1- anti-apoptotic protein, and I-309--chemokine-induced on the human endothelium by Lp(a)	/	[74]
UAP	Plasma	Olink Target 96 Cardiovascular III panel	AGRP, TGM2, IL6, GH1, and CA5A	MASCOT database	[75]
UAP	Plasma	MALDI-TOF MS	Antitrypsin, Fibrinogen gamma chain, and apolipoprotein A	MASCOT database	[76]

### 5.1 Proteomics in SAP

Proteomic analysis identified eight kinds of protein biomarkers in peripheral serum for the differentiation of patients with SAP, which were implicated in endothelial dysfunction and tissue protection/repair mechanisms [66]. Apart from proteins in the peripheral serum, proteins in platelets have the potential to become candidates for the efficient diagnosis of SAP. After analyzing the proteome of peripheral platelets, 18 types of differentially expressed proteins related to SAP were identified, which were enriched in cytoskeleton rearrangement, energy metabolism, oxidative stress, and protein degradation [67, 68]. Moreover, 206 different proteins that effectively differentiated patients with ST-segment elevation myocardial infarction and SAP were successfully identified by quantifying the proteins on the surface of percutaneous coronary intervention balloons [69]. A combination of proteomic analysis and western blotting indicated apolipoprotein A1 as a novel SAP biomarker [70]. In addition, proteomic analysis contributes to discriminating SAP from other cardiovascular diseases, such as AMI. A previous study revealed that peripheral serum proteins, including fibrinogen, fetuin-B, keratin 9, pro-apolipoprotein, and fibrinogen, were effective biomarkers for distinguishing SAP from AMI [71].

### 5.2 Proteomics in UAP

Differential proteins between patients with UAP and healthy individuals, such as hepcidin-25, MRP8, SAA, B2M, MPO, and βTG, were reported to be predominantly involved in vascular inflammation response, lipid metabolism disturbance, and atherogenic plaque stability from serum protein profiles [72]. A reduction in the level of serum complement C1q, which was related to the chronic inflammatory response, was identified as the dominant clinical indicator for the diagnosis of UAP through proteomic profiling analysis [73]. Plaque echogenicity combined with biomarkers identified using a protein microarray were used to accurately evaluate atherogenic plaque stability, which identified patients with UAP [74]. Moreover, multiple bioinformatics analysis approaches based on 25 different plasma proteins determined by proteomics suggested that proteins related to the inflammatory and AKT cascade responses, including AGRP, TGM2, and IL-6, which were prospective biomarkers for the demarcation of UAP and AMI [75]. More interestingly, different subtypes of plasma proteins, such as antitrypsin, fibrinogen gamma chain, and apolipoprotein A, were confirmed to contribute to differentiating patients with UAP from those with AMI [76].

## 6. Metabolomics

Metabolomics, positioned most downstream within the systems biology framework based on multi-omics, acts as a bridge connecting genotypes and phenotypes, reflecting the changes in genotypes and protein levels through alterations of metabolites [77]. Metabolomics aims to determine the relationship between metabolites and pathological changes by simultaneously conducting qualitative and quantitative analyses of all metabolites with low molecular weights in a specific pathological period in cells or organisms [78]. In contrast to the first three omics approaches, metabolomics highly reflects the environment in which cells or organisms are located, which is closely related to drug action, cellular nutritional status, and external environmental factors, hence, metabolomics has been widely employed in the fields of new drug research and development, disease diagnosis, and personalized treatment [79-81]. Metabolomic analysis approaches primarily include non-targeted and targeted metabolomics. Non-targeted metabolomics aims to maximally analyze metabolites from cells, bodily fluids, or tissue samples by adopting mass spectrometry technology and screening potential biomarkers for disease diagnosis [82]. Targeted metabolomics aims to determine the absolute content of specific metabolites in metabolic pathways of interest to verify the reliability of biomarkers [83]. Therefore, in this review, we focused on the application of non-targeted metabolomics to identify biomarkers of SAP and UAP. Potential biomarkers for identifying different subtypes and therapeutic targets of AP in metabolomic studies are summarized in Table 4.

### 6.1. Metabolomics in SAP

The alterations of multiple metabolic pathways, such as phospholipid catabolism, amino acid metabolism, and bile acid metabolism, are one of the characterizations of SAP [84]. Functional metabolomics studies have revealed that a significant differential metabolite related to SAP, N-acetylneuraminic acid, activates the Rho-associated coiled-coil-containing protein kinase signaling pathway by combining with RhoA and Cdc42 to aggravate myocardial injury [85]. Moreover, disturbances in valine, leucine, isoleucine, and glycerophospholipid metabolism have been confirmed as significant metabolic inducers of SAP [86, 87]. Higher levels of gut bacterial metabolites detected in stool samples, such as trimethylamine N-oxide, propionate, and butyrate, were also identified as SAP biomarkers [88]. Comprehensive consideration of threonine, threonine I, lactate dehydrogenase, and creatinine levels in the plasma has been reported as an effective method for differentiating SAP, with high accuracy [89]. Metabolomic evaluation of plasma samples suggested that elevated cystathionine levels induced the elevation of glucose, branched-chain amino acids, and phenylalanine and the decline of glutathione and pyridoxal phosphate, which were the dominant changes in SAP [90]. In addition, three phospholipid metabolites in plasma samples determined by mass spectrometry, namely lysophosphatidylcholine, phosphatidylcholine, and sphingomyelin, have been reported as potential biomarkers for discriminating SAP [91]. The dramatic increase in plasma metabolites such as trimethylamine oxide, choline, creatinine, and carnitine constitutes a novel diagnostic basis for SAP [92].

### 6.2. Metabolomics in UAP

Five metabolites, namely ceramide, glycocholic acid, allocholic acid, lithocholic acid, and leukotriene B4, have been suggested as potential metabolic markers of UAP [93]. Various metabolites identified by metabolomic analysis are enriched in multiple metabolic pathways, including arginine and proline metabolism, energy metabolism, glycerophospholipid metabolism, fatty acid metabolism, steroid hormone biosynthetic metabolism, and purine metabolism, which contribute to the clinical diagnosis of UAP [60, 94]. Nuclear magnetic resonance analysis was used to screen 17 types of metabolites that contribute to steroid hormone biosynthesis, aminoacyl-tRNA biosynthesis, and lysine degradation for the accurate diagnosis of UAP [95]. In addition, multiple metabolites or lipid species related to the glycerophospholipid, cysteine, and methionine metabolic pathways have been comprehensively adopted to effectively differentiate UAP [15]. The combination of the levels of differential plasma metabolites, such as specific ceramides, acylcarnitines, and fatty acids, with multiple machine learning (ML) approaches is an effective approach for UAP diagnosis [96, 97]. Furthermore, among the 19 types of metabolites identified using various analytical approaches, hydrogen sulfide was the most prominent endogenous biomarker for the determination of UAP [98].

**Table 4:** Potential biomarkers or therapeutic targets for identifying different AP subtypes in metabolomics studies.

AP subtypes	Samples	Platforms	Biomarkers/therapeutic targets	Datasets	Refs
SAP	Plasma	6530 Quadrupole-Time of Flight system	89 differential metabolites related to the reduction of phospholipid catabolism, tricarboxylic acid cycle, and biosynthesis of primary bile acid and the increase of amino acid metabolism and short-chain acylcarnitines	KEGG, MetaboAnalyst, Human Metabolome Database, and METLIN	[84]
SAP	Plasma	6530 Q/TOF-MS	*N*-acetylneuraminic acid	/	[85]
SAP	Venous blood	LC-ESI-MS/MS	Anserine, cytidine 5'-diphosphocholine, and 7,8-dihydro-L-biopterin	KEGG database	[86]
SAP	Plasma	UHPLC-QTOF/MS	Oxidized phospholipid and isoprostanes and isomers of prostaglandins	Metaboanalyst	[87]
SAP	Serum	^1^H NMR	Threonine, threonine I, lactate dehydrogenase, and creatinine	Human Metabolome Database	[89]
SAP	Plasma	LC-MS, GC-MS, HLPC, and NMR	Cystathionine induced elevation of glucose, branched-chain amino acids, and phenylalanine, as well as the decline of glutathione and pyridoxal phosphate	Human Metabolome Database	[90]
SAP	Serum	6540 UHD Accurate-Mass QTOFhybrid mass spectrometer	Lysophosphatidylcholine, phosphatidylcholine, and sphingomyelin	Human Metabolome Database	[91]
SAP	Serum	AB 4000 Triple Quadrupole Mass Spectrometer	Trimetlylamine oxide, choline, creatinine, and carnitine	/	[92]
UAP	Serum	LC-MS/MS3200Q1	Ceramide, glycocholic acid, allocholic acid, lithocholic acid, and leukotriene B4	/	[93]
UAP	Plasma	UPLC combined with Q-TOF/MS	Free fatty acids, amino acids, lysoPE and lysoPC species, and organic acids	KEGG and Human Metabolome Databases	[94]
UAP	Plasma	^1^H NMR	17 kinds of metabolites related to steroid hormone biosynthesis, aminoacyl-tRNA biosynthesis, and lysine degradation	Human Metabolome Database	[95]
UAP	Plasma	UPLC-ESI-MS/MS	72 and 88 metabolites/lipid species related to glycerophospholipid, cysteine, and methionine metabolism pathways	KEGG and MetaboAnalyst Databases	[15]
UAP	Plasma	UPLC/MS	LySoPC (18:0), 9-cis-Retinoic acid, Dehydrophytosphingosine, N-Acetyl-leukotriene E4, 4-Hydroxy-6-docosanone, 3-Methyl-2-butene-1-thiol, and L-Threonine	MetaboAnalyst	[97]
UAP	Blood	GC/MS, SPME-GC/MS, and ^1^H-NMR	Hydrogen sulfide	KEGG database	[98]

## 7. Integration methods for multi-omics technologies

Independent analysis of individual omics data may ignore the interactions between different omics levels during AP occurrence and development. A comprehensive understanding of the complex etiology and biological mechanisms of AP can be achieved by combining multiple omics data, such as genomics, transcriptomics, proteomics, and metabolomics. However, with the comprehensive application of multi-omics technologies, massive high-dimensional omics data pose challenges regarding their effective integration. Therefore, an urgent need for more effective algorithms, such as ML-based multi-omics integration methods, exists to capture potential relationships among multi-omics data. ML, as a branch of artificial intelligence, acquires potential and meaningful rules from a mass of data using diverse algorithms, including unsupervised and supervised ML approaches [99].

### 7.1. Unsupervised machine learning approaches

Unsupervised ML approaches identify hidden rules in data by analyzing their structure, patterns, and distribution, which are applied for clustering and dimensionality reduction, including k-means clustering, hierarchical cluster analysis, and principal component analysis (PCA) [100]. The subtypes, potential biomarkers, and mechanisms of AP have been identified by unsupervised analysis of protein or metabolite data, particularly via PCA application, contributing to its early diagnosis and treatment.

PCA was used to analyze proteomic data in the plasma of patients with AMI and ST elevations, those with SAP, those with UAP, and normal individuals, effectively distinguishing the acute coronary syndrome group (AMI and UAP) from the other groups (SAP and control) according to Principle Component 1. The acute coronary syndrome group was further separated into two discrete groups (AMI and UAP), according to Principal Component 2 [70]. The PCA loading matrix was established for 18 biomarkers related to the coronary flow velocity reserve in patients with SAP, identifying eight biomarkers with loadings >0.25, among which CCL16, CXCL16, PGLYRP1, TNFR1, GDF15, and TNFRSF10C were directly or indirectly associated with the pro-inflammatory hypothesis [101]. Metabolomics revealed 21 metabolites between the mini swine model and the sham operation group, 20 metabolites between patients with UAP and healthy people, and 8 shared metabolites between the swine model and patients with UA, based on which PCA effectively distinguished the swine model group from the sham operation group and patients with UA from healthy individuals [102].

### 7.2. Supervised machine learning approaches

Supervised ML approaches can predict unknown data by establishing a mapping relation based on input and output data, which are used to solve classification and regression problems, including support vector machines (SVM), artificial neural networks, decision trees, and random forests (RF) [103]. Supervised ML approaches are particularly suitable for the predictive diagnosis or classification of AP based on the omics data of existing patients, particularly for SVM and RF applications.

SVM was used to classify patient groups on training data based on proteomic data, and quadratic SVM was further adopted to distinguish patient groups on test data, revealing that six proteins discriminated significantly between SAP and low-risk control groups [104]. Multivariate linear SVM predicted seven differentially expressed metabolites, including LySoPC (18:0), 9-cis-Retinoic acid, Dehydrophytosphingosine, N-Acetyl-leukotriene E4, 4-Hydroxy-6-docosanone, 3-Methyl-2-butene-1-thiol, and L-threonine, as potential candidates for in-hospital patient outcomes [97]. Previous studies using RF have established a multiclass diagnostic model based on differential metabolites among patients with SAP or UAP, patients with AMI, and healthy controls. Six metabolites, including testosterone isobutyrate, N-acetyl-tryptophan, d-fructose, l-glutamic acid, erythritol, and gluconic acid, were core metabolites in the multiclass diagnostic model for SAP, AMI, and normal patients [105]. Meanwhile, four metabolites, 2-ketobutyric acid, LysoPC (18:2(9Z,12Z)), argininosuccinic acid, and cyclic GMP, were pivotal in the multiclass diagnostic model for UAP, AMI, and normal patients [106].

## 8. Discussions

**Figure 1.**
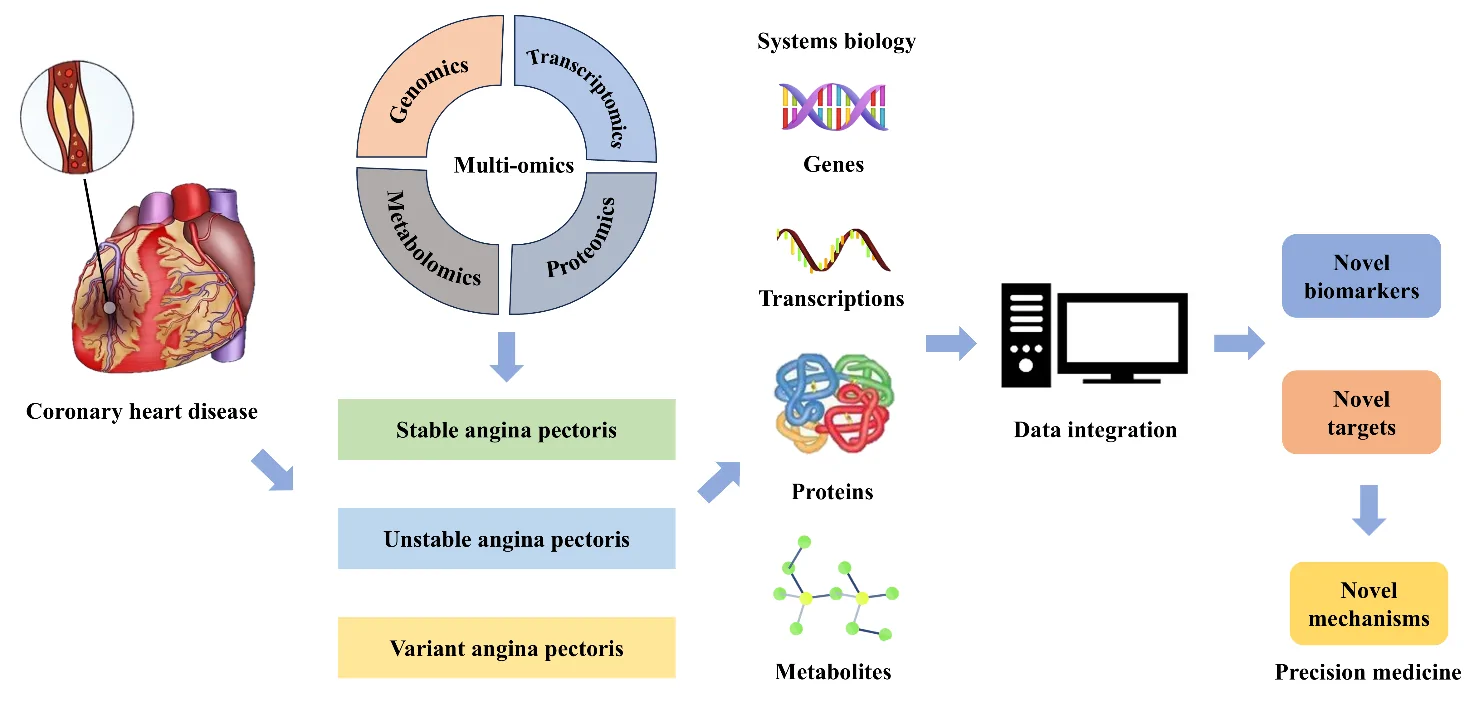
**The application of multi-omics technologies for AP classification and treatment**. Multi-omics technologies, namely genomics, transcriptomics, proteomics, and metabolomics, were applied to trace comprehensive and multidimensional biological information, including genes, mRNAs, proteins, and metabolites. After that, the biological information was processed by integration approaches to identify novel biomarkers and targets for AP classification and discover novel mechanisms for AP treatment, achieving precision medicine of AP.

AP, an important clinical symptom of CHD, is generally caused by an imbalance between the demand and supply of myocardial oxygen [107]. It may develop into AMI that seriously endangers the physical and mental health of patients if AP persists and cannot be alleviated promptly. Due to the various pathological mechanisms and therapeutic schedules of SAP, UAP, and VAP, an urgent need exists to employ efficient approaches to diagnose AP in a timely and accurate manner, achieving precision medicine. The emergence of omics technologies has effectively overcome this bottleneck. In contrast to traditional diagnostic methods for AP classification (including medical history inquiry, myocardial injury marker detection, and electrocardiogram and coronary angiography examinations), multi-omics technologies, namely genomics, transcriptomics, proteomics, and metabolomics, contribute to improving the sensitivity and specificity of diagnosis and achieving personalized diagnosis and precision medicine by tracing comprehensive and multidimensional biological information in AP’s different pathological stages from the level of genes, mRNAs, proteins, and metabolites (Fig. 1).

Previous studies have indicated that the pathological processes of AP vary in different individuals and are closely related to genetic factors [108]. Genetic variation is the main source of genetic risk for various diseases. Genomics applications have contributed to the accurate diagnosis and classification of AP by identifying genetic variations and polymorphisms. Genetic variations inducing AP can be rapidly and accurately screened from thousands of genes using multiple sequencing approaches, such as DNA microarray analysis, high-throughput sequencing analysis, and Sanger sequencing analysis [43]. A previous study using cDNA microarray analysis screened 482 variant genes in plaques from patients with SAP or UAP and identified five differential genes between these patient groups, including anticoagulant protein S, cyclooxygenase-1, interleukin-7, and chemokine monocyte chemotactic protein-1 and -2 [109]. In addition, GWAS can identify multiple susceptibility sites in genes based on sequenced genomes, greatly promoting genetic research in AP [110]. For example, a GWAS based on patient DNA microarray analysis demonstrated that homozygous carriers of the *rs112735431* site in RNF213 variants were associated with VAP [111]. RNF213 is located on human chromosome 17 and is mainly expressed in cells involved in vascular development and function, such as endothelial and smooth muscle cells [112]. As the main pathogenesis of VAP is spasms induced by dysfunction of coronary artery smooth muscle cells, the genetic variation of the RNF213 gene possesses the potential to become a clinically diagnostic tool for VAP. In addition, the variant distribution of bases in the *rs2066853* locus of the AhR gene, namely the combined genotype (GA + AA), contributes to the separation of different subtypes of CHD, including SAP and UAP [113]. As the gene polymorphism of AhR impacts immune/inflammatory response and UAP often accompanies stronger local inflammatory reactions than SAP, the gene polymorphism of AhR is expected to become a clinical classification tool for SAP and UAP [114]. The detected genetic variations and gene polymorphisms based on genomics are conducive to diagnosing and classifying AP, whose early intervention may be an effective strategy for avoiding serious cardiovascular events. Meanwhile, genomics application helps to predict the drug reactions of AP patients. The genomic analysis on cytochrome YP2C19 possible to predict the metabolic rate of UAP patients towards antiplatelet drugs, guiding dosage adjustments to reduce side effects and improve treatment efficacy [115].

Transcriptomics systematically analyzes the expression of all RNAs in the entire transcriptome, providing the possibility of screening differentially expressed RNAs for different AP subtypes and proposing novel therapeutic strategies. A previous study effectively identified 36 and 161 dysregulated miRNAs in SAP patients and UAP patients compared with normal ones by using the small RNA sequencing approach to establish circulating miRNA expression profiles, which served as potential biomarkers of SAP and UAP, respectively [60]. A previous study identified 94 differentially expressed miRNAs and 537 differentially expressed mRNAs between patients with UAP and healthy individuals by constructing the RNA expression of peripheral blood nuclear cells, which further screened the miR-4685-3p/C1QC signaling pathway to provide a novel therapeutic strategy for UAP [116]. MiR-4685-3p affects complement system activity, indirectly impacting the inflammatory response by regulating C1QC and vascular endothelial cell function to impact plaque stability by regulating MMP9 [117, 118]. Therefore, miR-4685-3p may be an effective tool for the clinical diagnosis of UAP. However, the traditional bulk RNA sequencing approach demonstrates the average expression level of total mRNA, which does not provide a detailed exploration of the RNA level in a specific cell [119]. In recent years, single-cell RNA sequencing technologies have been used to evaluate cell heterogeneity with unprecedented resolution and accuracy, identify new cell states and populations, elucidate dynamic cell transitions during development and differentiation, and capture specific changes in mRNA levels of cells, greatly improving our understanding of the pathogenesis and discrimination between different AP subtypes [120]. A previous study adopting single-cell RNA sequencing technology demonstrated the heterogeneity of immune cells in coronary plaques of patients with SAP, revealing the changes in cell types and characteristics of cell subgroups of immune cells in patients with SAP and identifying specifically high expression of the MCH-I signaling pathway in the T-cell cluster of patients with SAP [121]. Therefore, we believe that single-cell RNA sequencing technology has extensive prospects for understanding and classifying AP and other multiple cardiovascular diseases owing to its ability to reveal cellular heterogeneity, explore molecular mechanisms, and demonstrate dynamic processes, contributing to the discovery of novel biomarkers and personalized treatment strategies.

Since the development of proteomics, comparative studies have been performed on serum samples of AP to quantify overall protein expression levels, contributing to the identification of differential biomarkers of AP and judging its progression [66]. A previous study using LC-MS/MS identified 206 differentially abundant proteins that were captured on percutaneous coronary intervention balloons between patients with ST-segment-elevation myocardial infarction and SAP, which may be considered biomarkers because they were uniquely identified in patients with SAP [69]. Apart from screening biomarkers for classifying AP, proteomics identifies individual differences among different patients with the same AP subtype, achieving personalized therapy. A previous study screened 92 biomarkers from blood samples of female patients with AP using proteomics, among which the expression of GDF15 and vWF among all female patients differed, suggesting that treatment of these patients should be personalized [122]. GDF15, a stress factor, is involved in regulating acute and chronic inflammatory responses, whereas vWF, a glycoprotein secreted by endothelial cells and macrophages, promotes platelet aggregation and thrombus formation [123, 124]. We considered GDF15 and vWF to be effective clinical diagnostic tools for AP owing to the significant roles of the inflammatory response, platelet aggregation, and thrombus formation in AP pathogenesis.

In contrast to genomics, transcriptomics, and proteomics, the application of metabolomics in clinical practice is more easily accepted since it involves investigating changes in endogenous small-molecule metabolites in blood or urine via a non-invasive manner [125, 126]. The application of metabolomics in AP classification focuses on the identification of differential metabolites and their related pathways. A previous study performing metabolomic analysis on blood samples indicated that six core metabolites (testosterone isobutyrate, N-acetyl-tryptophan, d-fructose, l-glutamic acid, erythritol, and gluconic acid) enriched in the glucagon signaling pathway and d-amino acid and pyruvate metabolism pathways were conducive to diagnosing SAP [105]. Abnormalities in the glucagon signaling pathway and d-amino acid and pyruvate metabolism pathways affect blood glucose levels and the metabolic demands of the heart, indirectly exacerbating the heart’s hypoxic state [127, 128]. Therefore, we believe that these six core metabolites have the potential to be developed into tools for the clinical diagnosis of SAP. A previous study comparing differential metabolites in urine samples found that 16 metabolites enriched in amino acid and energy metabolism, fatty acid metabolism, and purine and steroid hormone biosynthetic metabolism pathways benefitted the clinical diagnosis of UAP [129]. Abnormal amino acid and energy metabolism may cause metabolic acidosis and exacerbate myocardial hypoxia, abnormal fatty acid metabolism may cause elevation of blood lipid levels and plaque and thrombi formation, and abnormal purine and steroid hormone biosynthetic metabolism may lead to insufficient energy supply, accumulation of calcium ions, inflammatory response exacerbation, and vascular constriction [130]. Although metabolomics has gradually made outstanding progress in the diagnosis of AP because of its unique advantages, some shortcomings are dominantly manifested in the following aspects [131]. First, the high requirements and complex procedures of metabolomics technology and the interference of external factors with metabolites may lead to instability of the quantified results [132]. For instance, cross-contamination with external environments and equipment should be avoided in the analysis of the gut microbiota and its metabolites to prevent changes in the microbiota and reduce the degradation of metabolites [133]. Furthermore, the metabolic pathways involved in discriminating different subtypes of AP by applying metabolomics remain in the exploratory stage, particularly for VAP, requiring a large number of samples and multicenter clinical and animal model experiments to demonstrate the practicality of metabolomics in mechanism exploration and subtype classification of AP [134]. The combination of metabolomics research with other omics investigating congenital etiology, such as genomics, transcriptomics, and proteomics, fully reflects the overall characteristics of the biological system and effectively compensates for the above-mentioned limitations of metabolomics.

**Figure 2.**
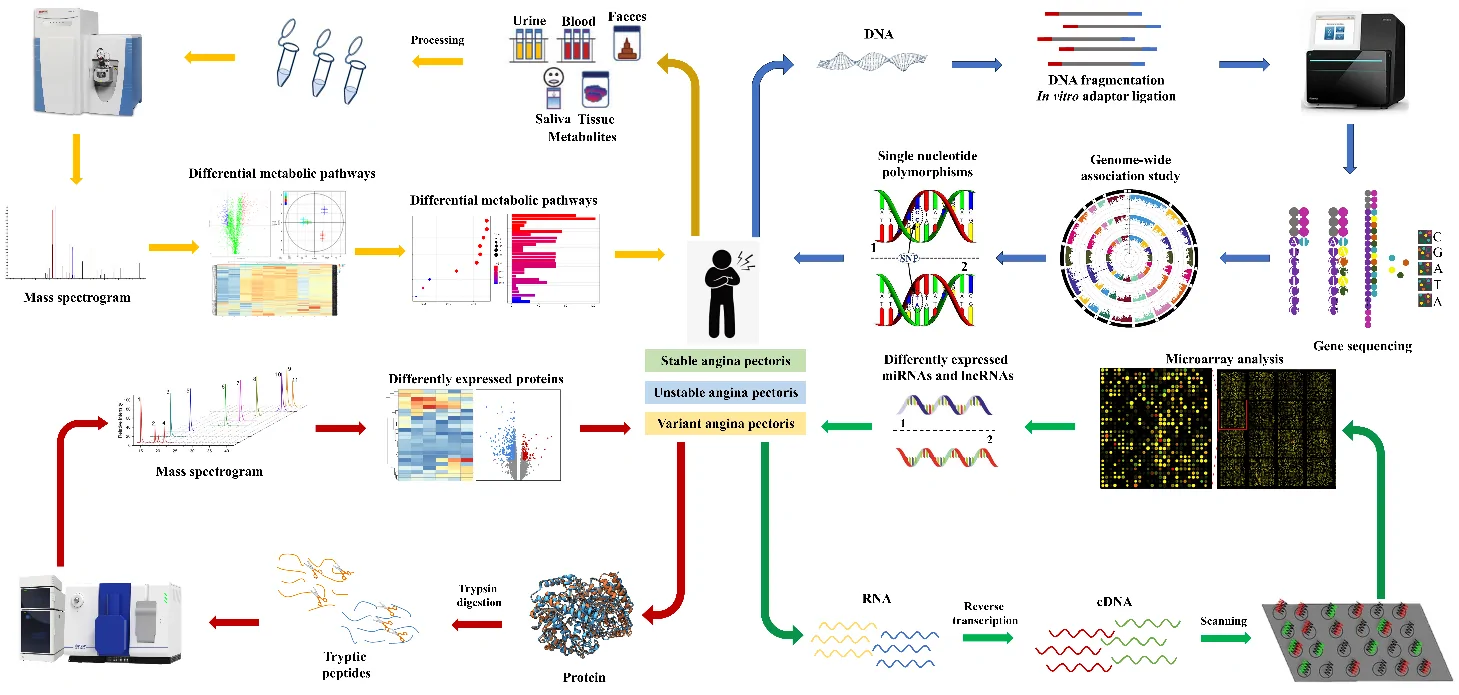
**Flow chart for applying genomics, transcriptomics, proteomics, and metabolomics in SAP, UAP, and VAP**. After obtaining genes, RNAs, proteins, and metabolites from patients with SAP, UAP, and VAP, DNAs and RNAs were sequenced, and proteins and metabolites were analyzed by mass spectrometry. After gene and RNA sequencing, the genetic variation and SNPs of DNA and differentially expressed miRNAs and lncRNAs in SAP, UAP, and VAP were screened. Differentially expressed proteins, differential metabolites, and metabolic pathways among SAP, UAP, and VAP were identified based on the mass spectrogram.

So far, we discovered that the existing studies mostly adopted the single omics approach to identify biomarkers and determine therapeutic targets and mechanisms of AP, whose application flow of single omics approach in SAP, UAP, and VAP has been presented in Figure 2. Although the application of omics technology is indispensable in the diagnosis and treatment of AP, it still has some limitations. The most important limitation is that single-omics studies frequently cannot provide comprehensive biological information, which may mask the interactions between molecules during AP progression. Specific RNA-mediated gene silencing occurs during the pathological process of AP, blocking gene expression at transcriptional and translational levels [135]. Therefore, single genomics was unable to identify silenced genes, while the combination of genomics and transcriptomics effectively screened the silenced genes, clarifying the pathological processes of AP. Moreover, proteins are not direct expression products of genes owing to multiple modifications post-translation, including phos-phorylation, acetylation, glycosylation, and ubiquitination [136]. For instance, glycosylated hemoglobin and hemoglobin are both transcribed and translated from the α-globin gene and β-globin gene [137]. However, after translation, hemoglobin is glycosylated to form glycosylated hemoglobin, which is a marker for predicting the severity of coronary artery disease and early outcomes in patients with SAP [138, 139]. Therefore, proteomics should be supplemented with genomics and transcriptomics to accurately classify AP. In addition, endogenous small-molecule metabolites detected using metabolomics compensate for the deficiency in proteomics caused by protein concentration, activity, and compensation effects [140]. Therefore, integrating multi-omics approaches is crucial to offer a more comprehensive understanding of AP. Certainly, although multi-omics studies possess great potential in revealing the complexity of biological systems and disease mechanisms, they also face a series of challenges, including the complexity of data integration and analysis, limitations of technology and platforms, and high costs. Therefore, effective algorithms and unsupervised and supervised ML approaches should be applied to multi-omics technologies.

A previous study had similar opinions to ours, suggesting that the integrated analysis of multi-omics approaches would provide helpful insights into ischemic stroke (IS) pathogenesis, therapeutic target identification, and biomarker discovery to achieve precision medicine for IS [139]. However, we found that the samples of multi-omics technologies applied to IS originated from clinical blood or urine and brain tissue of experimental animals. The samples of multi-omics technologies applied for AP originated only from clinical blood or urine, which is mainly attributed to the inability to fully replicate the clinical symptoms and multifactorial pathological processes of AP in experimental animals because they cannot experience and exhibit AP symptoms similar to humans. It follows that, based on existing clinical conditions and the precipitate development of omics technologies, the joint analysis of multiple omics, including genomics, transcriptomics, proteomics, and metabolomics, is particularly important for AP research, revealing the pathological mechanisms and progression of AP from multiple dimensions, thereby achieving precision medicine for AP.

Moreover, screened biomarkers of multi-omics techniques can guide clinical interventions for AP. Multi-omics technologies for SAP effectively identified genetic variation related to the metabolism and synthesis of cholesterol (such as apolipoprotein E and proprotein convertase subtilisin/kexin type 9), changes in gene expression related to lipid metabolism (such as sterol-regulatory element binding proteins and liver X receptors), proteins related to cholesterol metabolism and lipoprotein transport (such as apolipoprotein A1 and apolipoprotein B), and metabolites related to lipid and amino acid metabolism (such as phosphatidylcholine and tyrosine) [141-145]. Similarly, multi-omics technologies for UAP effectively identified gene polymorphism related to plaque stability [such as MMP1-1607dupG (rs1799750) and MMP3-1171dupA (rs3025058)], changes in gene expression (such as tissue plasminogen activator inhibitor) and proteins (such as soluble vascular cell adhesion molecule-1) related to plaque rupture and thrombosis formation, and metabolites related to inflammatory response (such as hs-CRP, TNF-α, and IL-6) [65, 146, 147]. Finally, multi-omics technologies for VAP effectively identified genetic factors that cause coronary artery spasms (such as Rho-associated kinase 2 polymorphism), changes in gene and protein expression (such as Rho-associated coiled-coil containing protein kinase), and metabolites (such as nitric oxide and nitrite) related to vascular smooth muscle contraction [148-150]. Clinical interventions can be modified over time based on the different genes, proteins, and metabolites detected. For example, statins, such as atorvastatin, should be administered if genes, proteins, and metabolites related to the metabolism and synthesis of lipids and cholesterol are screened in patients with AP. Antiplatelet and anticoagulant agents, such as aspirin and warfarin, should be administered if genes, proteins, and metabolites related to plaque rupture and thrombus formation are identified in patients with AP. Calcium channel antagonists, such as verapamil, should be administered if the genes, proteins, and metabolites related to vascular smooth muscle contraction are identified in patients with AP. Certainly, the biomarkers, therapeutic targets, and mechanisms are dynamically changing owing to the complex etiology of AP progression. Therefore, multiomics technologies should be applied to accurately identify biomarkers and therapeutic targets to adjust clinical treatment strategies in a timely manner, thereby achieving precision medicine for AP.

In addition, although multi-omics technologies have achieved AP classification by searching for biomarkers, such as differential genes, miRNA, proteins, and metabolites, they remain extremely insufficient for the understanding and treatment of AP. The screened biomarkers might originate from diverse cell types, which could conceal critical information in some cells indicating AP development, such as white blood cells and smooth muscle cells. Therefore, an urgent need to apply single-cell multi-omics exists to reveal the cellular heterogeneity and microscopic mechanisms of AP through molecular data at the single-cell level, achieving more precise treatment decisions and effective personalized treatment plans. Moreover, samples for existing omics technologies applied to AP classification were mostly serum and urine, which do not involve the lesion site. Therefore, we believe that future applications of spatial multi-omics may contribute to revealing the interactions between different cell types and changes in the local microenvironment at the lesion site by obtaining spatial location information of cells at the single-tissue level, aiding in revealing the spatial heterogeneity of AP and comprehensively understanding the changes in spatial structure during AP development. In addition, most previous studies have focused on comparing the omics differences between patients with AP and normal patients at a specific moment. However, as we know, AP development is a dynamic process of change. Future applications of spatiotemporal multi-omics may effectively reveal dynamic changes in coronary artery plaques, endothelial cells, immune response-related genes, transcriptomes, proteins, and metabolites during the different stages of AP to provide comprehensive and accurate molecular spectra, contributing to revealing the spatiotemporal heterogeneity of AP and understanding its dynamic changes to achieve precision medicine. We believe that multi-omics technology will provide a clearer blueprint for accurately diagnosing and treating the three AP subtypes if these prospects are successfully achieved.
